# Can handheld ultrasound probes reliably measure transabdominal prostate and bladder volumes? A prospective randomized point-of-care ultrasound study

**DOI:** 10.3389/fruro.2024.1362734

**Published:** 2025-02-11

**Authors:** Henry C. Wright, Dillon Corrigan, Smita De

**Affiliations:** ^1^ Northwestern Medicine Department of Urology, Chicago, IL, United States; ^2^ Cleveland Clinic, Quantitative Health Sciences, Cleveland, OH, United States; ^3^ Cleveland Clinic, Glickman Urological and Kidney Institute, Cleveland, OH, United States

**Keywords:** benign prostatic hyperplasia, BPH, ultrasound, point-of-care ultrasound (POCUS), handheld ultrasound

## Abstract

**Background:**

National guidelines recommend obtaining prostate gland volume (PGV) prior to benign prostate hyperplasia (BPH) surgery. Measurement of PGV with handheld ultrasound (HUS) probes shows promise.

**Objective:**

To compare the reliability of two HUS probes (Butterfly iQ and Clarius C3) to the BPH guideline-recommended imaging (GIm) for both prostate and bladder volumetrics.

**Methods:**

Male patients with GIm were randomized to undergo transabdominal HUS PGV with one of the two probes. A subset underwent voided volume measurements with one of the two HUS and a conventional bladder scanner (BS). The reliability of the volume measurements was assessed for each probe via intraclass correlation coefficients (ICCs). We utilized the following standard criteria: ICC < 0.5: poor reliability; 0.5 ≤ ICC < 0.75: moderate reliability; and ICC ≥ 0.75: good reliability.

**Results:**

A total of 78 men in the prostate arm (38 Butterfly, 40 Clarius) and 45 in the bladder arm (24 Butterfly, 21 Clarius) were randomized and included in this study. The mean prostate volume based on GIm was larger in the Clarius group (*p* = 0.044). Other baseline characteristics were similar between groups (*p* > 0.05). The ICCs were 0.78 (95% CI: 0.62, 0.88) and 0.71 (95% CI: 0.51, 0.83) for the Butterfly and Clarius probes, respectively. Regarding bladder volumetrics, the ICCs were 0.82 (95% CI: 0.19, 0.95), 0.72 (95% CI: 0.44, 0.88), and 0.69 (95% CI: 0.13, 0.87) for the Butterfly, Clarius, and bladder scanner, respectively.

**Conclusions:**

The Butterfly iQ demonstrated good reliability for PGV and voided volume measurements, in comparison to moderate reliability for Clarius C3.

## 1 Introduction

Prostate gland volume (PGV) is useful for the management of patients with benign prostatic hyperplasia (BPH), lower urinary tract symptoms (LUTS), and other prostate disorders. The American Urological Association (AUA) and European Urological Association (EAU) BPH guidelines recommend PGV assessment prior to surgical intervention (1, 2). As per the guidelines, this volume assessment may be obtained via transabdominal ultrasound (TAUS), transrectal ultrasound (TRUS), computed tomography (CT), or magnetic resonance imaging (MRI), henceforth referred to as guideline-recommended imaging (GIm). However, TRUS is invasive and uncomfortable, CT requires ionizing radiation, and MRI is relatively expensive and may not be readily available at the time of clinical assessment. In addition to the PGV assessment, the guidelines also recommend the assessment of post-void residual (PVR) for patients with LUTS prior to the decision for BPH surgical intervention.

**Figure 1:**
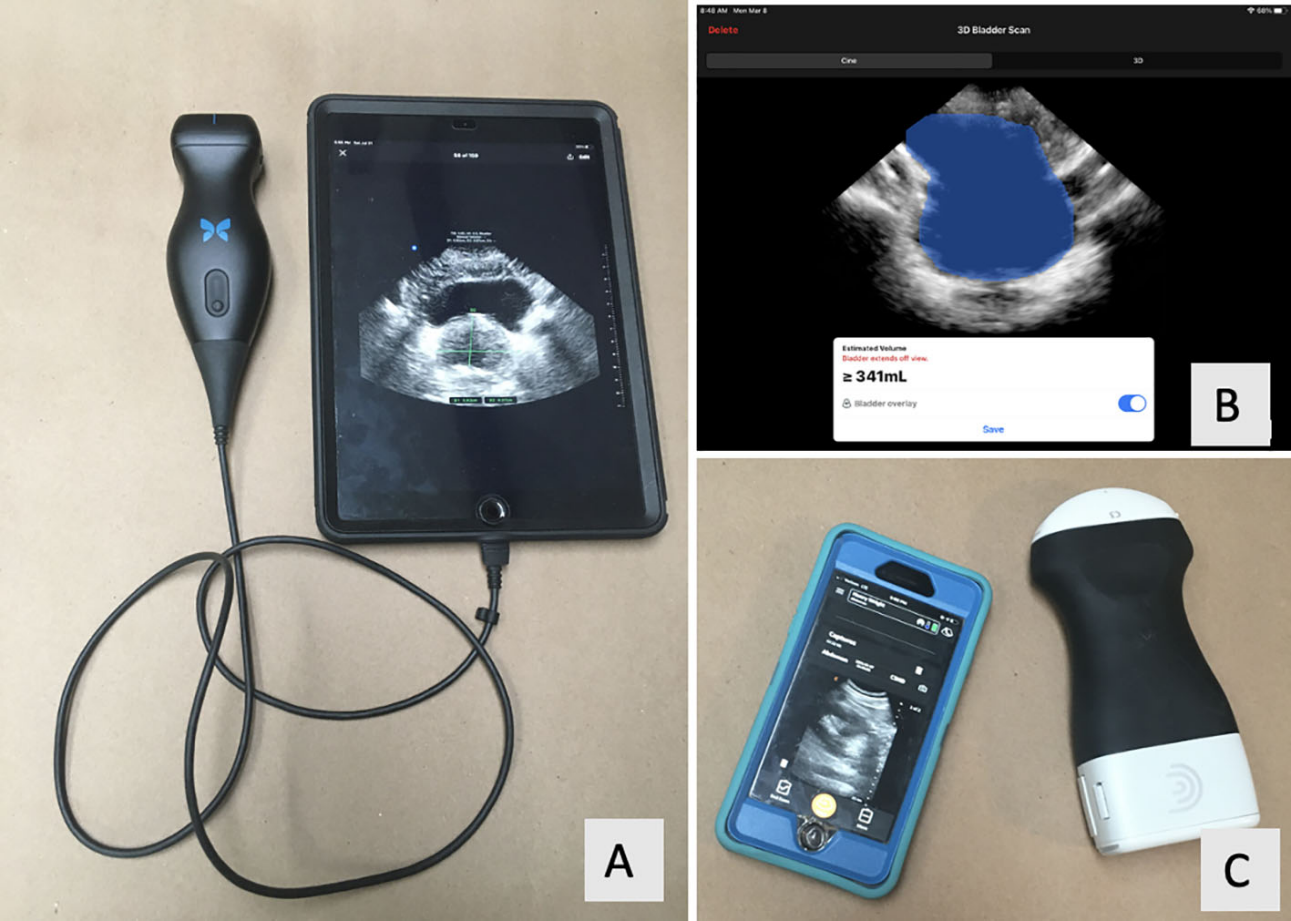
**(A)** Butterfly IQ probe (Guilford, CT) with the Apple iPad (Cupertino, CA). **(B)** Screenshot of the Butterfly IQ’s bladder scanning function. **(C)** Clarius C3 probe (Vancouver, BC) with Apple iPhone.

There has been great progress with ultrasound technology, particularly with its use in endourology. Handheld ultrasound (HUS) probes are recently developed devices that directly connect to smartphones or tablet computers to display images (**Figure 1**). In comparison to conventional US machines, HUS probes are growing in popularity because they are more affordable and user-friendly and can easily fit into one’s pocket. Most utilize piezoelectric ceramics for image generation, similar to conventional ultrasound machines. Other HUS probes produce images with a digital chip, which is a new technology in the world of ultrasound (3). Included with the HUS probes are software applications (apps) for image assessment such as measuring, labeling, and user-friendly artificial intelligence (AI) functions to automatically calculate bladder volumes such as PVR. To our knowledge, there are no studies that evaluate HUS probes as a means for point-of-care ultrasound (POCUS) for both prostate and bladder volumetrics in the endourology clinic as compared to GIm.

Our primary objective was to evaluate and assess the reliability of HUS in measuring PGV compared to the “gold standard” reference GIm (TRUS, TAUS, CT, or MRI). The secondary objective was to evaluate and assess the reliability of HUS and a dedicated bladder scanner (BS) machine (Echonous, Redman, WA) in measuring bladder volumes to calculate and compare to actual voided urine volume. The specific HUS probes used for this study were the Butterfly iQ (Guilford, CT), which incorporates a digital chip to generate and receive the ultrasound signal, and the Clarius C3 (Vancouver, BC), which utilizes standard piezoelectrics. We hypothesize that measurements obtained by both HUS probes will reliably measure PGV compared to GIm despite differences in technology (i.e., digital chip in Butterfly iQ), limited image manipulation, and smaller screens with the HUS probes (as compared to standard US machines).

## 2 Materials and methods

In this prospective, randomized trial, adult male patients were recruited from a high-volume urology clinic. Eligible patients must have undergone GIm of the prostate within 12 months of enrollment without interval prostate surgery. We chose 12 months because the prostate volume does not significantly change during this time frame (4, 5). Patients were excluded if they had a history of prostatectomy, were unable to be supine for 30 min, were unable to get on an exam table without assistance, were unable to give consent, or had a body mass index (BMI) >50 kg/m^2^ as higher BMI can impair ultrasound image quality (6). We calculated a target sample size of 40 patients per arm based on a previous similar study that examined 36 patients with an in-office POCUS (11), using a 5% margin of error and 95% confidence interval. Enrolled patients were randomized in a 1:1 ratio using a computer-generated randomization scheme to undergo transabdominal HUS with one of the two HUS probes. All enrolled patients underwent HUS prostate volumetrics. A subset of patients underwent pre- and post-void bladder volumetrics. Institutional review board approval was obtained (IRB #20-644).

### 2.1 The ultrasound procedure

#### 2.1.1 Prostate volumetrics

Patients were placed in the supine position on a clinic exam table. They were asked not to void prior to the exam in order to 1) have a partially distended bladder to enhance prostate imaging (7) and 2) allow for pre- and post-void bladder measurements, as described below. An ultrasound gel was applied and the prostate was identified by placing the probe midline just above the pubic bone and angling it caudally. Prostate measurements were obtained at the widest intervals in the following dimensions: width (axial view, lateral to lateral), height (axial view, posterior to anterior), and length (sagittal view, base to apex). Intravesical protrusion of the prostate was noted as present or not present. One fully trained urologist experienced in US image acquisition and US-guided genitourinary procedures and trained to use the HUS probes performed all the HUS procedures. This urologist was blinded to any prior prostate imaging results and did not participate in the GIm prostate measurements or interpretations.

Prior review articles demonstrated that the accuracy of MRI is superior to TRUS/TAUS, which is superior to CT (8). Therefore, if a patient had multiple GIm available, we selected the comparison based on that order.

TRUS was performed by fully trained urologists blinded to the HUS results. Prostate dimensions acquired via CT were measured by a fully trained urologist (HB) blinded to the HUS results. A second fully trained urologist (FH) also assessed prostate dimensions via CT. If there was more than a 10-g discrepancy between the two urologists, then a third urologist (SD) was used as the tiebreaker. MRI- or TAUS-based prostate measurements were obtained from the radiology interpretation report. An Apple (Cupertino, CA) iPad 7th generation was used to display all HUS images. All PGV measurements were calculated with the ellipsoid formula (height × width × length × π/6). While other formulas have been utilized, the ellipsoid is commonly used for its simplicity and accuracy, particularly for larger glands (9).

#### 2.1.2 Bladder volumetrics

During the same session as prostate volumetrics, pre- and post-void bladder volumes were obtained in a subgroup of patients. Patients were included in this subgroup if there was a reason for a change in bladder volume at the time of data acquisition, whether it be spontaneously voiding or needing catheter insertion (henceforth considered “void”). Pre- and post-void bladder volumes were calculated by the HUS app’s AI bladder volume measuring tool, which utilizes a simple “point and measure” function. For comparison, patients underwent pre- and post-void bladder measurements with a conventional BS. This study’s BS has a screen to confirm bladder targeting and AI software to automatically calculate bladder volume. The same BS machine was used for all patients and is routinely calibrated for daily use in the urology clinic. Patients voided into a volume-marked urinal to document voided volume. This voided volume was used as the gold standard for bladder voided volumetrics. Therefore, the pre- and post-void volumes for two scenarios (Butterfly and BS or Clarius and BS) were used to calculate a measured “voided volume,” which was the test variable. BS was performed by urology nurses, all trained to use the device. HUS was performed by the same urologist performing HUS prostate volumetrics. BS and HUS were performed immediately before and after the participant voided and within 2 min of each other, and each user was blinded to the other’s results.

#### 2.1.3 Data analysis

We collected the following variables: patient demographics, urologic surgical and medical history, prostate medication use [alpha blocker or 5-alpha reductase inhibitor (5ARI)], dates of guideline and HUS imaging, prostate dimensions, pre- and post-void bladder volumes (and voided volume via the difference of the two), actual voided volume (via volume marked urinal), duration of ultrasound session for both bladder and prostate imaging (probe on skin to probe off skin), presence of intravesical prostatic protrusion, and costs of the devices.

Agreement between HUS and GIm was assessed via Bland–Altman (BA) analysis. The differences in volume measurements were assessed for normality and, if they were not approximately normally distributed, were re-expressed on the logarithmic scale (10). Limits of agreement (LoAs) between the two methods were calculated as the average difference ± 1.96 * standard deviation (SD) of the differences. LoAs calculated on the log scale were back-transformed to the original scale as previously described (11). Reliability was quantified by estimating the intraclass correlation coefficient (ICC) and its 95% confidence interval. The form of the ICCs calculated in this study is based on a two-way random effects model, assessing absolute agreement. We implemented the following classifications of reliability: ICC < 0.5: poor reliability, ICC ≥ 0.5 or < 0.75: moderate reliability, and ≥0.75: good reliability (12).

All tests were considered significant at the *p <*0.05 level. All analyses and graphics were performed using the R statistical package 4.1.0. Randomization protocol and study data were collected and managed via our institutional RedCap (Research Electronic Data Capture) database.

## 3 Results

### 3.1 Prostate volumetrics

**Figure 2:**
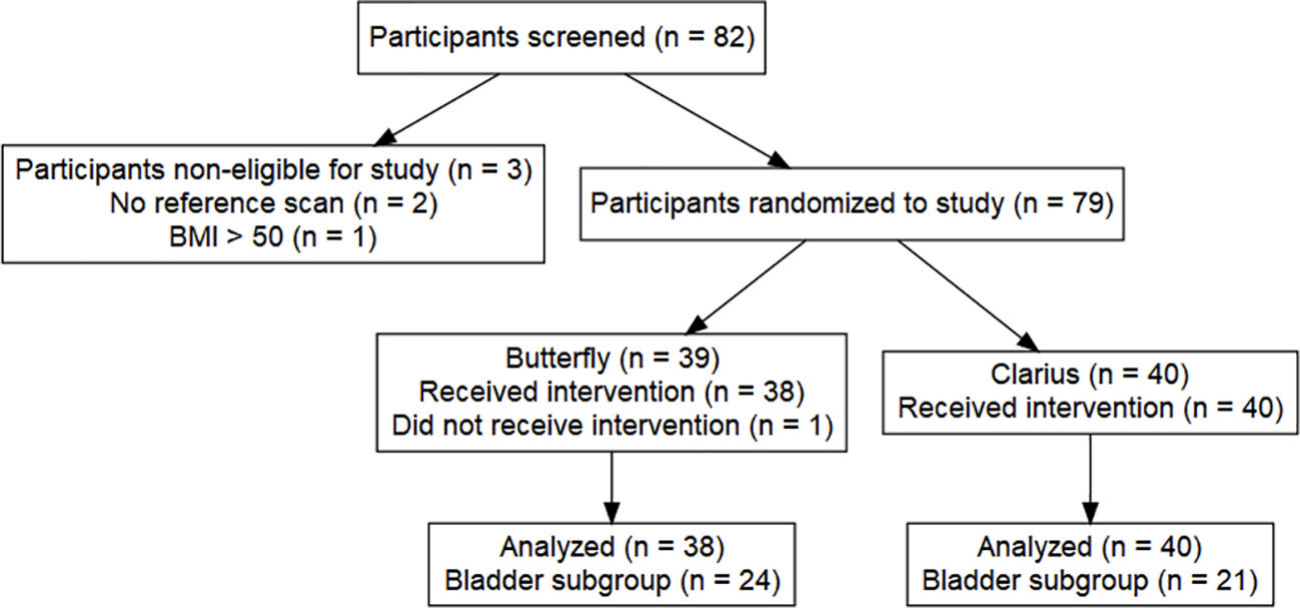
Flowchart of study design.

A total of 82 patients met the inclusion criteria and were approached for study enrollment from September 2020 to March 2021. Three patients did not complete the study requirements due to failure to obtain GIm (*n* = 2) and having an actual BMI >50 despite having a BMI <50 at screening (*n* = 1). All enrolled patients gave informed consent. A total of 79 patients were enrolled (13). In the Butterfly arm, one subject was unable to complete all the HUS imaging and, thus, was not included in the final analysis, resulting in a total of 78 patients with a complete PGV dataset (38 Butterfly, 40 Clarius, **Figure 2**).

Baseline demographics, presenting symptoms, and urologic history were similar between groups (**Table 1**, *p* > 0.05). The mean prostate size measured by the Butterfly probe was 47 g, compared to 79 g for Clarius, which was significantly different (*p* = 0.011, **Table 1**). Despite randomization, the GIm-measured prostate volume in the Butterfly arm was 41 g, compared to 62 g for Clarius (*p* = 0.044, **Table 1**). The mean duration between guideline imaging and HUS imaging was 92.5 days, with no difference between the Butterfly and Clarius groups (*p* = 0.61). Mean imaging time with the Clarius probe was longer than with the Butterfly, at 102 and 68 s, respectively, yet this was not significant (**Table 1**, *p* = 0.07). A total of 22 (27.8%) patients with intravesical prostatic protrusion were identified (11 Butterfly, 11 Clarius).

**Table 1:** Baseline characteristics for prostate volumetric patients.

Characteristic	Butterfly, *N* = 38^1^	Clarius, *N* = 40^1^	*p*-value^2^
**Age**	67 (59, 74)	68 (60, 76)	0.4
**Race**			>0.9
Black or African American	7 (18%)	7 (18%)	
White	31 (82%)	33 (82%)	
**BMI**	27.1 (25.8, 30.0)	27.1 (25.2, 31.0)	>0.9
**Prior BPH surgery**	3 (7.9%)	7 (18%)	0.4
**Other prior urologic surgery**	19 (50%)	14 (35%)	0.3
Presenting symptom			
LUTS	15 (39%)	15 (38%)	>0.9
Urinary retention	6 (16%)	13 (32%)	0.15
Kidney stones	22 (58%)	23 (57%)	>0.9
Hematuria	3 (7.9%)	4 (10%)	>0.9
**Alpha blocker use**	23 (61%)	26 (65%)	0.9
**5ARI use**	12 (32%)	13 (32%)	>0.9
**Prostate HUS session length (s)**	68 (41, 123)	102 (54, 188)	0.072
**Prostate volume, HUS (g)**	47 (29, 81)	79 (43, 113)	0.011
**Prostate volume, GIm (g)**	41 (30, 81)	62 (38, 112)	0.044
**Guideline imaging**			0.736
MRI	6 (15.8%)	2 (5.0%)	
TRUS	9 (23.7%)	8 (20.0%)	
TAUS	3 (7.9%)	2 (5.0%)	
CT	20 (52.6%)	28 (70.0%)	

BMI, body mass index; LUTS, lower urinary tract symptoms; BPH, benign prostatic hyperplasia; 5ARI, 5-alpha reductase inhibitor; OAB, overactive bladder; MRI, magnetic resonance imaging; TRUS, transrectal ultrasound; TAUS, transabdominal ultrasound; CT, computed tomography.

^1^Median (IQR); *n* (%).

^2^Kruskal–Wallis rank sum test; Pearson’s chi-squared test.

**Figure 3:**
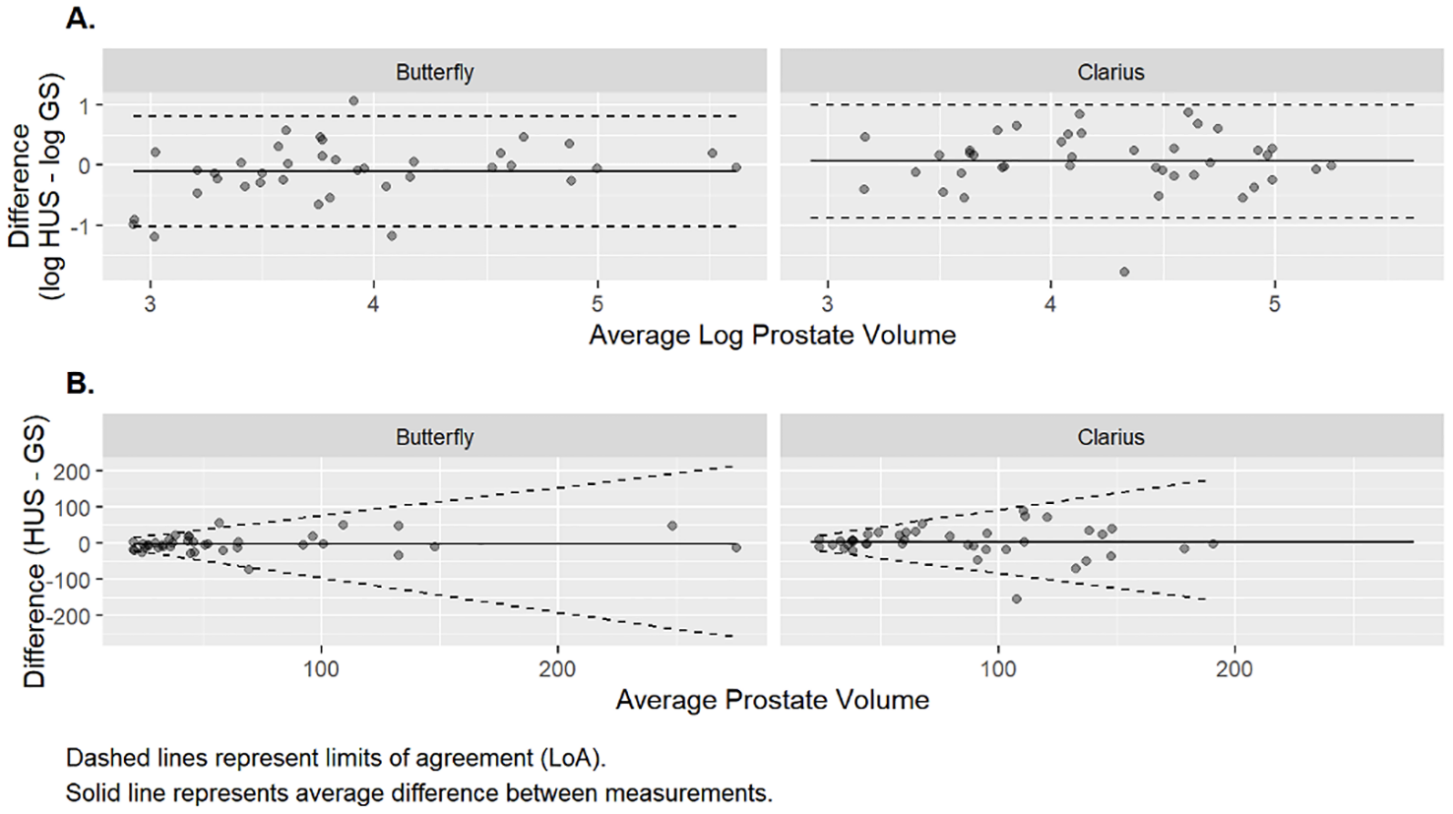
Bland-altman analysis of prostate volume measurements.

The prostate volumetric ICCs were 0.78 (95% CI: 0.62, 0.88) and 0.71 (95% CI: 0.51, 0.83) for the Butterfly and Clarius probes, respectively (**Table 2**). This indicates good reliability for the Butterfly HUS and moderate reliability for the Clarius HUS. The BA plot also shows smaller bias and narrower limits of agreement for the Butterfly iQ than the Clarius C3 (**Figure 3**).

**Table 2:** Reliability between HUS and reference measurements.

Imaging method	ICC^1^	95% CI
Prostate cohort		
Butterfly	0.78	(0.62, 0.88)
Clarius	0.71	(0.51, 0.83)
Bladder cohort		
Butterfly	0.82	(0.19, 0.95)
Clarius	0.72	(0.44, 0.88)
Bladder scanner	0.69	(0.13, 0.87)

^1^ICC < 0.5: poor reliability; 0.5 ≤ ICC < 0.75: moderate reliability; and ICC ≥ 0.75: good reliability.

### 3.2 Bladder volumetrics

**Figure 4:**
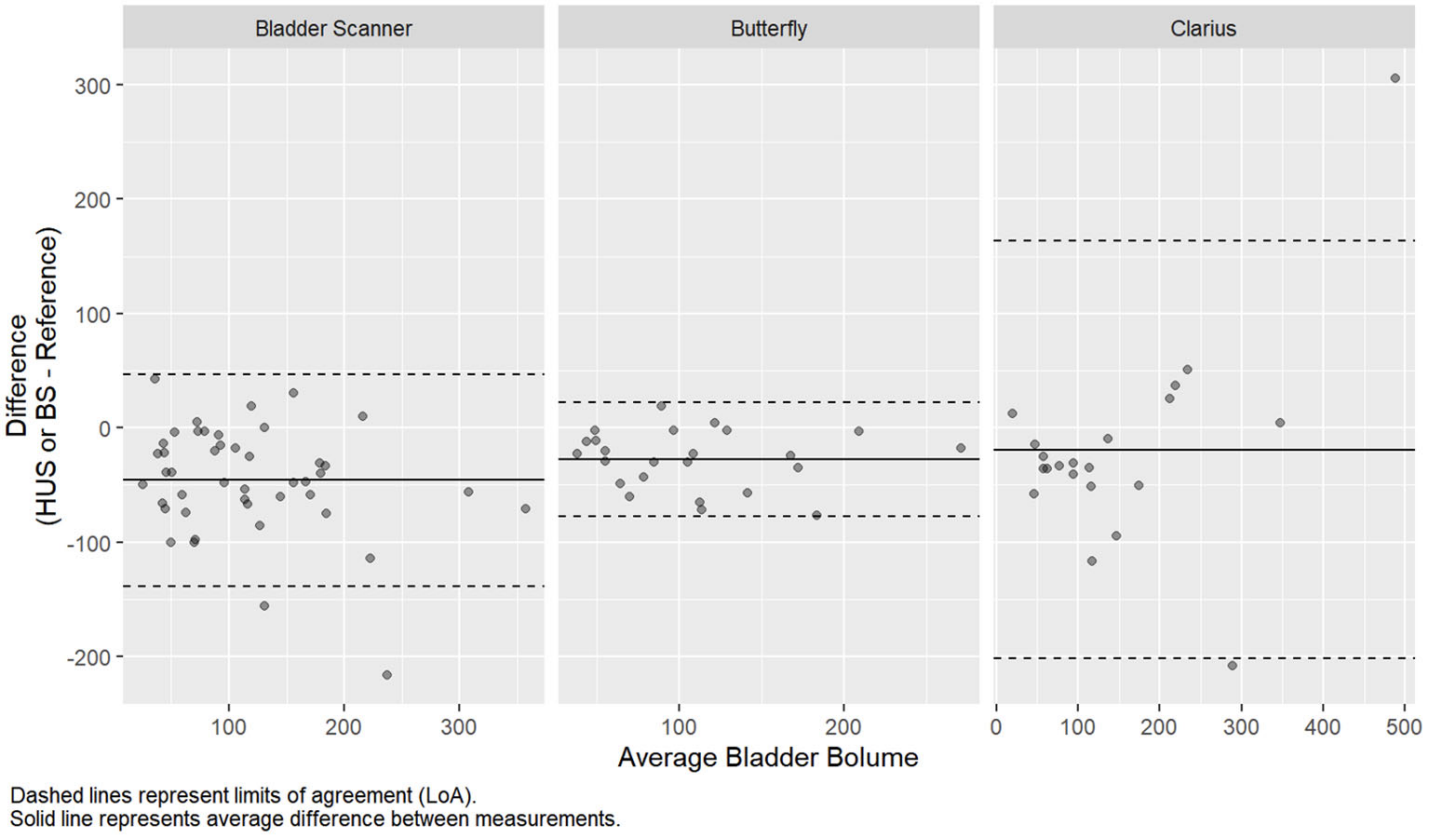
Bland-altman analysis of bladder volume measurements.

A subset of 45 patients underwent pre- and post-void bladder volumetrics (24 Butterfly, 21 Clarius). Baseline demographics, presenting symptoms, and urologic history were similar between groups (**Table 3**, *p* > 0.05). The duration of the HUS session was shorter for Butterfly (14 s) compared to Clarius (27 s, *p* < 0.001). The average differences in volume measurements for the Butterfly, Clarius, and BS were −22.67 mL (95% CI: −77.64 mL, 22.31 mL), −19.27 mL (95% CI: −201.70 mL, 163.16 mL), and −45.91 mL (95% CI: −138.81 mL, 46.99 mL), respectively (**Table 4** and **Figure 4**).

**Table 3:** Baseline characteristics in bladder subgroup patients.

Characteristic	Butterfly, *N* = 24^1^	Clarius, *N* = 21^1^	*p*-value^2^
**Age (years)**	64 (54, 71)	65 (59, 73)	0.4
**Race**			>0.9
Black or African American	5 (21%)	4 (19%)	
White	19 (79%)	17 (81%)	
**BMI**	27 (26, 30)	27 (26, 32)	0.8
**Prior BPH surgery**	3 (12%)	6 (29%)	0.3
**Other prior urologic surgery**	13 (54%)	6 (29%)	0.2
Presenting symptom			
LUTS	10 (42%)	9 (43%)	>0.9
Urinary retention	2 (8.3%)	5 (24%)	0.3
Kidney stones	17 (71%)	12 (57%)	0.5
Hematuria	3 (12%)	2 (9.5%)	>0.9
**Alpha blocker use**	16 (67%)	13 (62%)	>0.9
**5ARI use**	4 (17%)	6 (29%)	0.5
**Bladder HUS session length (s)**	14 (12, 22)	27 (21, 36)	<0.001
**Voided volume, HUS (mL)**	85 (45, 125)	90 (45, 185)	0.6
**Voided volume, BS (mL)**	74 (27, 90)	115 (72, 160)	0.032
**Actual voided volume (mL)**	110 (78, 155)	141 (80, 200)	0.2

BMI, body mass index; LUTS, lower urinary tract symptoms; BPH, benign prostatic hyperplasia; 5ARI, 5-alpha reductase inhibitor; OAB, overactive bladder.

^1^Median (IQR); *n* (%).

^2^Kruskal–Wallis rank sum test; Pearson’s chi-squared test.

**Table 4:** Average differences and limits of agreement between HUS and reference measurements.

Imaging method	Average difference (SD)	LoA^1^	Average log difference (SD)	Log LoA^2^	LoA as a function of average X^3^
Prostate					
Butterfly	−0.92 g (24.67 g)	–	−0.1 g (0.47 g)	−1.03 to 0.82	−0.47*X* to 0.39X
Clarius	4.05 g (41.29 g)	–	0.07 g (0.48 g)	−0.88 to 1.01	−0.41*X* to 0.47X
Bladder					
Butterfly	−27.67 mL (25.5 mL)	−77.64 mL to 22.31 mL	–	–	–
Clarius	−19.27 mL (93.07 mL)	−201.7 mL to 163.16 mL	–	–	–
Bladder scanner	−45.91 mL (47.4 mL)	−138.81 mL to 46.99 mL	–	–	–

^1^Limits of agreement are average difference ± 1.96SD difference, based on original measurements.

^2^Limits of agreement are average difference ± 1.96SD difference, based on log-transformed measurements.

^3^Limits of agreement are back-transformed from log LoA, based on the methods described in Euser, Dekker, and le Cessie 2008.

The intraclass correlation coefficients were 0.82 (95% CI: 0.19, 0.95), 0.72 (95% CI: 0.44, 0.88), and 0.69 (95% CI: 0.13, 0.87) for the Butterfly, Clarius, and bladder scanner, respectively (**Table 2**). This indicates good reliability for the Butterfly HUS and moderate reliability for the Clarius HUS and BS for voided volume measurements. Regarding the costs of these HUS probes plus the dedicated tablet computer, the Butterfly iQ totaled $2,328, while the Clarius C3 totaled $5,229. The BS machine evaluated in this study cost $9,500.

## 4 Discussion

Ultrasound is a key part of the urologist’s armamentarium; however, there are many opportunities to increase its use in our daily practice. Here, we evaluate low-cost, recently developed portable HUS probes for use in the endourology clinic to assess prostate and bladder volumetrics. To the best of our knowledge, this study is the first of its kind to compare two different HUS probes to AUA and EAU BPH guideline-recommended imaging modalities for PGV assessment in a urology clinic. Our results demonstrate that the Butterfly iQ probe more reliably and accurately measured PGV and voided volume than the Clarius C3 probe. This is based on higher ICC and narrower limits of agreement on our statistical evaluation. We theorize that the ICC was more reliable for the Butterfly iQ probe for a few reasons: 1) clearer images as displayed on the iPad; 2) faster and more responsive software; and 3) while we cannot conclusively say this from a technical or physics perspective, the images were subjectively clearer for deeper structures (like prostate) on the Butterfly iQ compared to the Clarius C3. Both HUS probes outperformed the BS when predicting voided volume. Notably, there were wide confidence intervals for the bladder volume measurements that were likely related to the large range of actual voided volumes and our small sample size, and as such, results should be confirmed with larger studies.

We evaluated two HUS probes with differing technologies: the Clarius C3 employs the commonly used piezoelectrics, while the Butterfly iQ employs a novel digital chip to generate and receive ultrasound images (3). The advantages of a digital chip include reduced cost and wide adaptability. For example, the Butterfly iQ can function as a linear, curved array, and phased probe without needing different hand pieces. This technology appears promising and did not appear to have a negative effect on our study. In general, we found that both probes were easy to operate and we encountered minimal technical challenges with their use.

HUS probes demonstrate utility across several fields in medicine and surgery (14–18), yet evidence for their use in urology is currently emerging. Moussaoui et al. performed TAUS and TRUS with the Clarius C3 probe on 98 patients and compared the PGV measurements to prostatectomy specimen weight (19). They found a strong correlation for larger prostates only (>60 g) while using the ellipsoid formula for both TAUS and TRUS, which does not align with our results, as the HUS probes evaluated in our study had a stronger correlation with smaller glands. Notably, the methods between the two studies are different in terms of determining standard size (image-based volume vs. pathologic specimen weight), and mean prostate size was similar. The researchers also demonstrated the identification of an intravesical median lobe in 8.3% of their patients. Twenty-eight percent of the prostates imaged in our study were found to have intravesical prostatic protrusion. This finding is important for BPH surgical planning.

Another area where POCUS may be of utility is in the inpatient setting where they may provide a quick non-invasive result that can aid in rapid decision-making. Previously, Lavi et al. conducted a study on admitted patients undergoing bedside bladder and prostate diagnostics by comparing a urology trainee to an ultrasound technician with a HUS (Vscan, GE Healthcare, Chicago, IL) and a conventional US device (13). Although their sample size was small (25 prostates, 26 PVRs), they found no statistically significant difference between users or ultrasound devices. It is notable that the mean prostate volume in this study was 39.3 g, smaller than that found in our study, yet this is not surprising as many of our patients were being evaluated for BPH/LUTS and would likely have larger prostates than the average patient.

We believe these HUS devices show potential to be used as a POCUS for a variety of genitourinary disorders, yet the understanding of their true utility is in its early stages. Education is an essential aspect of broadening the implementation of these devices and familiarizing urologists on their use. Uy et al. developed and implemented a training program to teach Canadian urology trainees about POCUS (20). They demonstrated that participants significantly improved their understanding and confidence in performing POCUS with HUS probes. In our study, we found the HUS devices to be simple, user-friendly, and easy to learn, yet additional studies examining their reliability and utility are warranted.

This study has limitations. First, our study compared HUS to a variety of imaging modalities (MRI, TRUS, TAUS, CT). Each modality varies in their accuracy for measuring PGV (8), which does make direct comparisons challenging. However, the intent of this study was to be as close to the ”real world” as possible—we find most patients with prostate pathology present to the clinic with any or all of these modalities already performed and thus used for BPH surgical planning. Second, ultrasound has a known intra- and interuser variability, and while our study had one qualified urologist performing all HUS measurements, we did not control for the variability that may exist between users performing TRUS and radiology-performed TAUS and similarly cannot comment on generalizability based on this study alone. Third, approximately a third of our patients were on 5ARIs. While these medications reduce prostate volume, they do so over a period of up to 6–8 months. Since the mean duration between GIm and HUS prostate imaging was 92 days, we do not believe that the unlikely event of interval initiation of a 5ARI would have notably altered our findings. Fourth, the bladder subgroup was relatively small despite our best efforts to ask patients not to void prior to study enrollment (many had already voided or did not need to). Overall, we found the HUS bladder volume calculators quick and easy to use, with no notable technical difficulties and with the added advantage of producing a clear ultrasound image of the bladder. Finally, we did not tightly control pre-void bladder volumes, which may have impacted HUS imaging of the prostate (7), particularly for patients with decompressed bladders.

Despite these limitations, our study demonstrates that the HUS probes reliably produce PGV measurements compared to GIm, with the Butterfly iQ outperforming the Clarius C3. Additional studies are necessary to further evaluate the reliability and precision of these probes and to determine if these may potentially be considered a replacement option for GIm. At this time, we believe our data demonstrate that these HUS probes may be considered useful for supplemental imaging—to help with surgical planning (i.e., identifying intravesical prostatic protrusion or median lobes) and evaluating other genitourinary organs not assessed in this study. These HUS probes do show promise in calculating bladder volumes, such as PVR, but future studies with larger patient cohorts may be of benefit. Notably, HUS probes are less costly than the BS evaluated in this study and much less costly compared to CT, MRI, or traditional US machines and could possibly provide an opportunity for improved care in practices that may be in remote locations or with limited resources. We hope that this study and others like it continue to demonstrate the growing potential of these handheld probes as a new technology for point-of-care imaging for the practicing endourologist.

## Data Availability

The original contributions presented in the study are included in the article/supplementary material. Further inquiries can be directed to the corresponding author.
